# A Novel High-Content Flow Cytometric Method for Assessing the Viability and Damage of Rat Retinal Ganglion Cells

**DOI:** 10.1371/journal.pone.0033983

**Published:** 2012-03-23

**Authors:** Zhi-Yang Chang, Da-Wen Lu, Ming-Kung Yeh, Chiao-Hsi Chiang

**Affiliations:** 1 Graduate Institute of Life Sciences, National Defense Medical Center, Neihu, Taipei, Taiwan; 2 Department of Ophthalmology, Tri-Service General Hospital, National Defense Medical Center, Neihu, Taipei, Taiwan; 3 Institute of Preventive Medicine, National Defense Medical Center, Sanhsia, Taipei, Taiwan; 4 School of Pharmacy, National Defense Medical Center, Neihu, Taipei, Taiwan; University of Washington, United States of America

## Abstract

**Purpose:**

The aim of the study was to develop a high-content flow cytometric method for assessing the viability and damage of small, medium, and large retinal ganglion cells (RGCs) in N-methyl-D-aspartic acid (NMDA)-injury model.

**Methods/Results:**

Retinal toxicity was induced in rats by intravitreal injection of NMDA and RGCs were retrogradely labeled with Fluoro-Gold (FG). Seven days post-NMDA injection, flatmount and flow cytometric methods were used to evaluate RGCs. In addition, the RGC area diameter (D_(a)_) obtained from retinal flatmount imaging were plotted versus apparent volume diameter (D_(v)_) obtained from flow cytometry for the same cumulative cell number (sequentially from small to large RGCs) percentile (Q) to establish their relationship for accurately determining RGC sizes. Good correlation (r = 0.9718) was found between D_(a)_ and apparent D_(v)_. Both flatmount and flow cytometric analyses of RGCs showed that 40 mM NMDA significantly reduced the numbers of small and medium RGCs but not large RGCs. Additionally, flow cytometry showed that the geometric means of FG and thy-1 intensities in three types of RGCs decreased to 90.96±2.24% (P<0.05) and 91.78±1.89% (P>0.05) for small, 69.62±2.11% (P<0.01) and 69.07±2.98% (P<0.01) for medium, and 69.68±6.48% (P<0.05) and 69.91±6.23% (P<0.05) for large as compared with the normal RGCs.

**Conclusion:**

The established flow cytometric method provides high-content analysis for differential evaluation of RGC number and status and should be useful for the evaluation of various models of optic nerve injury and the effects of potential neuroprotective agents.

## Introduction

Retinal ganglion cells (RGCs) are neurons that receive visual information from photoreceptors via intermediate neurons and transmit messages to the brain. Several experimental models, including ischemia reperfusion, optic nerve injury, intravitreal excitatory amino acid injection and ocular hypertension, have been used to investigate pathogenic processes of RGCs [1]. A combination of retrograde labeling and retinal flatmount is frequently applied to quantify RGCs in intervention-induced RGC toxicity. Several neuronal tracers, such as fluoro-Gold (FG) [2], di-I (1, 1-dioctadecyl-3, 3, 3′, 3′-tetramethyl-indocarbocyanine perchlorate), and fast blue have been used to label RGCs [3]. FG is one of the most important tracing agents. After injecting the FG tracer into superior colliculi, the tracer is transported in a retrograde way through the optic nerve to obtain FG-labeled RGCs up to 85% [4]. Image-analysis software is then used to count the RGCs in a high-throughput and size-differentiated fashion [5], [6]. The FG-tracer method provides a reliable measurement to determine the number of RGCs, but no further information regarding the function or damage of RGCs is obtained. Additionally pattern electroretinography can be used for determining the function of RGCs *in* vivo, but the methodology is limited only qualitatively measuring the overall RGC function [7].

In rat, three different sizes of RGCs, including large, medium, and small RGCs, have been established. These correspond to alpha, beta, and gamma RGCs, respectively, in morphological classification [8], [9]. Despite the different characteristics of large, medium, and small RGCs, most investigations report RGC damage combining them together. This is primarily because a feasible and convenient method for separating three groups of RGCs to evaluate their damage independently is lacking. Thus, a quantitative method for rapidly evaluating the number and damage of large, medium, and small RGCs in pharmacological studies is highly desired.

Currently, high-content analytical technology is applied to evaluate multiple biochemical and morphological properties in a single cell. Flow cytometry has been used extensively in the study of high-content analysis. Flow cytometric signals provide rich information about cell features. For instances, forward scatter (FSC) correlates with cell volume; side scatter (SSC) corresponds to internal complexity; and the signals of fluorescence (FL) represent characters and intensities of fluorescent-labeled cells [10]. Although, flow cytometry has been applied for assessing the liability of rat RGCs [11], however, the method alone does not obtain additional information about the damage of survived RGCs. The goal of this study was to develop a flow cytometric method associated with biomarkers and neuronal tracers for assessing the viability and damage of small, medium, and large RGCs in an NMDA-induced rat retinal damage model. Thy-1 is primarily expressed by RGCs within the retina, some RGC stressors, including increased IOP [12], [13], optic nerve crush [12], [13], [14], ischemia [15], [16] and intravitreal injection of excitatory amino acid [12], [15], [16] have been shown to decrease the levels of thy-1 mRNA and protein in RGCs. The decrease in thy-1 mRNA and protein precedes and is greater than the RGC loss, suggesting that thy-1 is an early marker of RGC stress. [1], [12], [14]. In this study, thy-1 was used as a serrogate marker for RGC status. Retrograde transport of FG is related to the transporting ability of RGC axons [17], the intensity of the FG in RGCs was assayed to evaluate the damage status of RGC axons. The acquired data, FSC and different fluorescences of flow cytometry, were used to analyze the biophysical and biochemical features of RGCs.

## Methods

### Animals

Male Wistar rats (Taiwan National Laboratory Animal Center, Taipei, Taiwan) weighing between 225 and 250 g, were housed in a temperature-controlled (21–22°C) environment under a 12-h light-dark cycle. All studies were handled in accordance with the Association for Research in Vision and Ophthalmology Statement on the Use of Animals in Ophthalmic and Vision Research. The protocol was approved by the Institutional Animal Care and Use Committee of National Defense Medical Center (Permit number: IACUC-07-175 and IACUC-08-209).

### Drug treatment

Neurotoxicity was induced with NMDA (Sigma, St. Louis, MO) as previously reported [18]. Briefly, rats were anesthetized with a mixture of ketamine hydrochloride (50 mg/kg, Nang Kuang Pharmaceutical, Tainan, Taiwan) and xylazine hydrochloride (13.3 mg/kg, Sigma, St. Louis, MO). After the application of topical 0.5% proparacaine hydrochloride (Alcon Lab, Fort Worth, TX), an intravitreal injection of 2 µL NMDA (40 mM) prepared in BSS PLUS® solution (Alcon Lab, Fort Worth, TX) was performed in the right eye of each rat using a 30-gauge needle connected to a 10-µL microsyringe (Hamilton, Reno, NV). The solution was injected into the sclera at approximately 1 mm behind the limbus. The BSS PLUS® solution was used as a vehicle control and injected into the left eye.

### Retrograde labeling of retinal ganglion cells

RGCs were labeled with FG (Sigma, St. Louis, MO) by injecting the FG solution into the superior colliculi using a stereotaxic device (Stoelting, Wood Dale, IL) as described previously [19], [20]. Briefly, four days post-NMDA injection, rats were first anesthetized with the ketamine/xylazine mixture and the skin over the cranium was incised to expose the scalp. Two vertical holes, 1 mm in diameter, were drilled on both sides of the skull with a dentist's drill 6 mm posterior to the bregma and 1.5 mm lateral to the midline. Two microliter of 3% FG solution was delivered by using a micropipette at depths of 3.8, 4.0 and 4.2 mm from the bone surface.

### Retinal flatmount imaging and FG-labeled RGC counting

Seven days post-NMDA injection, the rats were euthanized with CO_2_ and the eyes were immediately enucleated. The retinas were dissected and fixed in 4% paraformaldehyde (Sigma, St. Louis, MO) for 1 hour. After phosphate buffered saline (PBS) washing, retinal flatmounts were prepared by making four radial incisions and placing the retinas on slides in 10% glycerol (Sigma, St. Louis, MO) in PBS. For RGC counts, the retinal slides were observed under a fluorescence microscope (Olympus BX-50, Olympus Optical, Tokyo, Japan) using UV excitation (330–385 nm) and a barrier filter (420 nm). Digital images were taken using a CCD camera (SPOT, Diagnostic Instruments, Sterling Heights, MI). Each retina was visually divided into four quadrants (superior, inferior, nasal and temporal). Quadrants were further divided into central (0.8–1.2 mm from the optic disc), middle (1.8–2.2 mm from the optic disc) and peripheral regions (0.8–1.2 mm from the retinal border). At each region, two fields (200×200 µm^2^) were counted. Two methods, either manual or automatic counts, were used to quantify RGCs. For automatic counts, the digital images were processed using Image J (http://rsbweb.nih.gov/nih-image/, U.S. National Institutes of Health, Bethesda, MD) and the RGB (red-green-blue) images were converted to 8-bit grayscale for binary counting [21]. The RGCs were classified into three groups based on soma sizes described previously (small: <9.4 µm; medium: 9.4–12.6 µm; and large: >12.6 µm) [8]. RGC density was expressed as the number of RGCs per square millimeter of the counted retina area.

### Double immunofluorescence labeling and terminal uridine deoxynucleotidyl transferase dUTP nick end labeling

To prepare retinal cell suspension, the dissected retinas were incubated in papain solution (contained 20 U/mL papain, 1 mM L-cystein, 0.5 mM EDTA and 200 U/mL DNase I [Sigma, St. Louis, MO] in Earle's balanced salt solution [EBSS, Invitrogen, Carlsbad, CA]) at 37°C for 40 minutes. The retinas were transferred to ovomucoid-BSA buffer (1 mg/mL ovomucoid, 1 mg/mL BSA, and 100 U/mL DNase I in EBSS) for 5 minutes at 37°C. Then, tissues were gently triturated through a plastic pipette until dispersed and the retinal cells were fixed with 2% paraformaldehyde in PBS for 20 minutes at room temperature. The cells were centrifuged and resuspended in PBS containing 0.4% triton X-100 (Sigma, St. Louis, MO) and 1% BSA was added to block unspecific binding of antibodies. Each sample was subsequently centrifuged and resuspended in DNA-labeling solution (APO-BrdU™ TUNEL Assay Kit; Invitrogen, Carlsbad, CA) for 1 hour at 37°C to label DNA strand breaks in apoptotic cells. The cells were then incubated with rabbit anti-Fluoro-Gold antibody (1∶100, Millipore, Bedford, MA) mouse peridinin-chlorophyll protein (PerCP)-labeled anti-Thy1.1 antibody (1∶100, BD Biosciences, Franklin Lakes, NJ) and FITC-labeled anti-BrdU antibody (1∶200, eBioscience, San Diego, CA) for 30 minutes. After washing with PBS, the cells were incubated with goat anti-rabbit IgG phycoerythrin-R (PE) conjugated antibody (1∶200, Santa Cruz Biotechnology, Santa Cruz, CA) for 30 minutes. The cells were then washed with PBS, resuspended in 1 mL PBS containing 0.1% triton X-100. The total number of retinal cells was counted using a hemacytometer (Bright-Line, Reichert, Buffalo, NY) and the size distributions of cells were evaluated via flow cytometry.

### Flow cytometry

Retinal cells were evaluated using a flow cytometer (Facscaliber; Becton Dickinson, San Jose, CA). The measurement conditions were optimized based on preliminary studies with following settings: FSC value for reflecting cell volume: voltage E00, amplifier gain 2.3; SSC value for reflecting cytoplasmic structure: voltage 400, amplifier gain 2.0; FL1 reflecting green labeled fluorescence intensity (FITC): voltage 350; FL2 reflecting labeled orange fluorescence intensity (PE): voltage 350; FL3 reflecting labeled red fluorescence intensity (PerCP): voltage 470; and flow rate: high. The compensation values determined from CaliBRITE three-color kit (BD Biosciences, Franklin Lakes, NJ; three beads each with one marker, FITC, PE or PerCP, separately; for assessing single color bead: each bead 20 µL wtih 980 µL PBS; the mixture of three color beads: 3×20 µL beads with 940 µL PBS) were set as follows. FL1–%FL2: 0.7%; FL2–%FL1: 20.0%; FL2–%FL3: 0.0%; and FL3–%FL2: 17.5%.

Cell sizes were estimated from the FSC signals using the calibration curve established by 6, 10, 15 and 20 µm-diameter of polystyrene microspheres (20 µL microsphere solution+980 µL PBS) (Polysciences, Warrington, PA). For each sample, 10,000 cells were counted automatically. Incubated cells without the fluorescence conjugated antibody served as the blank. FG intensity and Thy1.1 expression were evaluated as geometric means of FL2 and FL3 fluorescence intensities, respectively. The fluorescence intensity was determined and the results were processed by CellQuest Pro software (Becton Dickinson, San Jose, CA) and FCS Express V3 (De Novo Software, Los Angeles, CA).

### Correlation between flatmount imaging and flow cytometry data

RGC area diameters (D_(a)_) of control group were calculated from the area values of flatmount imaging and automatic counts. The projecting area (A) equation of a circle (*A = πD^2^/4*…eq.1) was used for the calculation. D_(a, Q)_ was defined as a area diameter for the cumulative cell number (sequentially from small to large RGCs) percentile (Q) of the determined RGCs with size under the diameter. D_(a, Q)_ values of RGCs in control group for Q at 5, 20, 40, 60, 80, and 95% were obtained from the data of flatmount imaging and automatic counts. Apparent volume diameters (D_(v)_) of RGCs were estimated from the FSC values determined from the flow cytometer by substituting these values into the established calibration curve of the standard microspheres. Consequently, apparent D_(v, Q)_ values of control group for Q at 5, 20, 40, 60, 80, and 95% were obtained. The relationship of D_(a, Q)_ and apparent D_(v, Q)_ was also analyzed.

### Statistical analysis

All results were expressed as mean ± standard error of the mean (SEM). The density and percentage of each RGC group in NMDA-injured and control groups were statistically analyzed by Student's *t*-test using SPSS 12 software for Windows (SPSS Inc., Chicago, IL). The level of significance was set at P<0.05.

## Results

### Retinal flatmount imaging and counting

**Figure 1:**
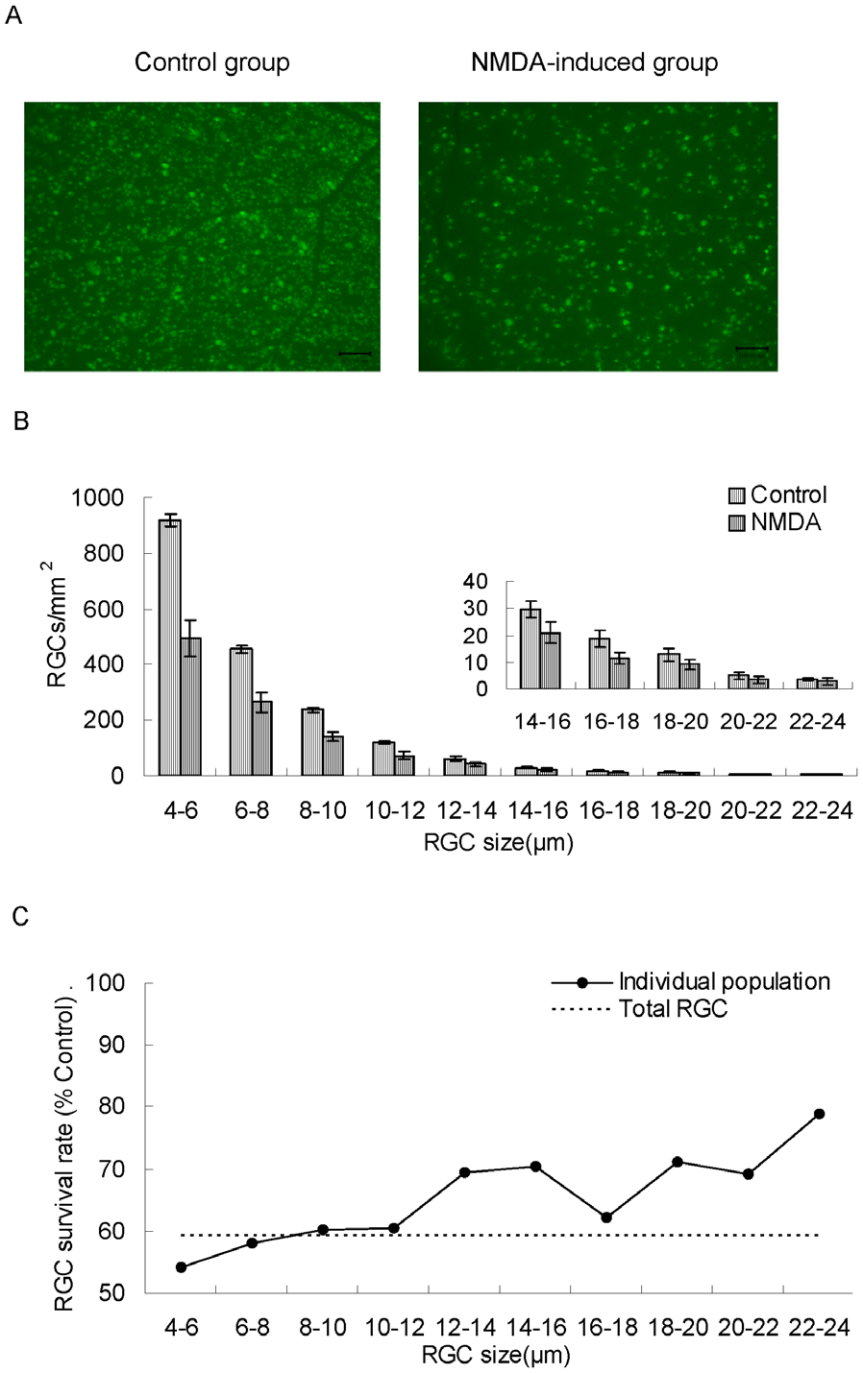
Flatmount quantification of rat RGCs by image analysis software. Seven days post-treatment with intravitreal injection of vehicle (BSS as control) or NMDA solution. (A). Image of FG-labeled RGCs in flatmounted retina, bar = 100 µm. (B). Soma size distribution histograms. The histograms were generated by counting 10000 RGC cells and dividing into size groups with a 2 µm interval. RGC density was expressed as RGCs/mm^2^ (mean ± SEM, n = 8). (C). RGC survival rates of different size groups after intravitreal NMDA (40 mM, 2 µL) treatment. Each point represents RGC survival rate (%) based on the individual RGC size population of control group, the dashed line represents total RGC survival rate (%).

FG-labeled RGCs were examined and counted (Fig. 1A). The quantifications of RGCs were obtained by image-analysis-software and manual methods. The density of RGCs in the control group obtained from two methods were similar (image-analysis-software and manual methods, 1866±34 cells/mm^2^ vs. 1933±28 cells/mm^2^, mean ± SEM, n = 8). The consistent results of the retrograde labeled RGCs were obtained with very good precision (coefficient of variation <5%) in two counting methods. The retinal area (estimated by Image J) was 59.45±1.57 mm^2^ (n = 8) associated with total RGC counts between 131,100 and 117,000 cells.

The size distribution of FG-labeled RGCs is shown in Fig. 1B. Mean RGC densities and normalized frequency of small, medium, and large RGCs were 1554±31, 195±4, and 117±14 cells/mm^2^ and 83.31±1.64%, 10.42±0.23% and 6.26±0.75%, respectively (n = 8, Table 1).

**Table 1:** Normalized frequency of rat RGCs in control and NMDA-induced groups.

Group	Normalized frequency (%)			
	Total RGCs	Large RGCs	Medium RGCs	Small RGCs
**Control**	100.00±1.84%	6.26±0.75%	10.42±0.23%	83.31±1.64%
**NMDA-induced**	59.33±6.76%‡	4.41±0.96%	6.43±0.99%†	46.74±5.92%‡

RGCs were classified as small, medium and large groups based on their soma sizes: small = <9.4 µm; medium = 9.4–12.6 µm; large = >12.6 µm (mean ± standard error of the mean, n = 8). The number of RGCs was normalized with total RGCs of control group. Control and NMDA-induced groups were statistically analyzed by the Student's *t*-test. *P<0.05,

† P<0.01 and

‡ P<0.001.

On the seventh day post-intravitreal NMDA injection, total RGC densities obtained from two counting approaches were 1107±126 cells/mm^2^ for the image-analysis-software method (59.34±6.76% of the control group, Table 1) and 1025±132 cells/mm^2^ for the manual method (53.03±6.83% of the control group) (P<0.001, n = 8). The survival rate of RGCs with diameter <10 µm was less than that of total RGCs (Fig. 1C). When evaluating RGC survival rates, flatmount analysis showed that the RGC densities of small and medium RGCs were significantly affected to be reduced to 872±110 and 120±19 cells/mm^2^ (46.74±5.92% and 6.43±0.99% of total RGCs of control group, P<0.01, n = 8) (Table 1), but the large RGCs seemed invulnerable to NMDA toxicity and no significant difference in RGC density was observed between the two groups.

### Preliminary analysis of flow cytometry

**Figure 2:**
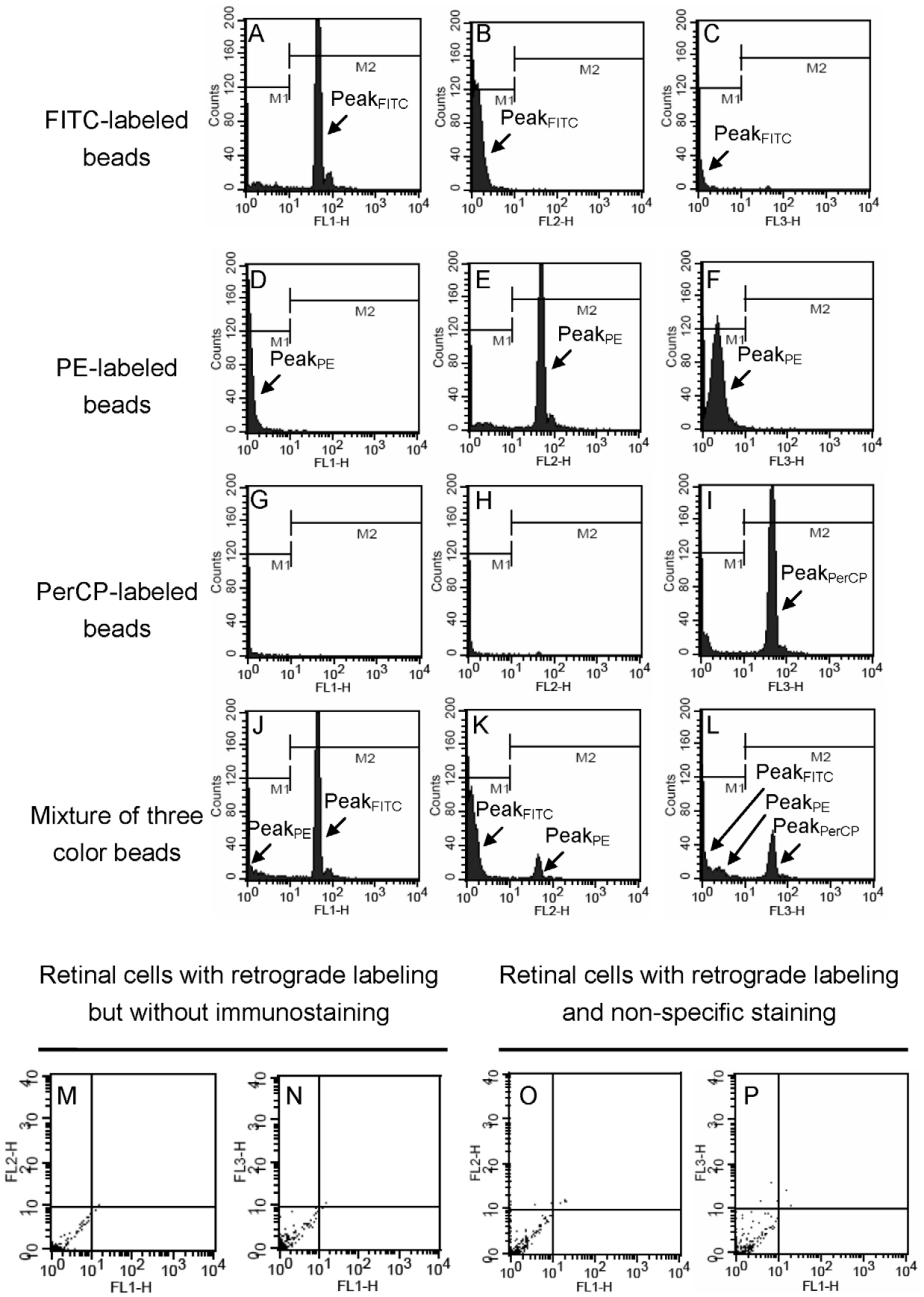
Preliminary analysis of flow cytometry. (A–L) Representative flow cytometric histograms of fluorescein isothiocyanate (FITC, Ex: 490 nm; Em: 520 nm)-labeled beads (A–C), phycoerythrin-R (PE, Ex: 495 nm; Em: 578 nm)-labeled beads (D–F), peridinin-chlorophyll protein (PerCP, Ex: 490 nm; Em: 677 nm)-labeled beads (G–I) and the equal-amount mixture of three color beads (J–L). The FL-1H histogram (A) shows the positive peak of FITC-labeled beads (peak_FITC_) (in region M2, intensity >10^1^); the FL-2H histogram (E) shows the positive peak of PE-labeled beads (peak_PE_); the FL-3H histogram (I) shows the positive peak of PerCP-labeled beads (peak_PerCP_). The FL-1H (J), FL-2H (K), and FL-3H (L) histograms of the mixture of three color beads show positive peaks of FITC, PE, and PerCP-labeled beads, respectively. (M–P) Representative flow cytometric dot plots of negative controls. >99.98% retinal cells with retrograde labeling but without immunostaining (M, N), and >99.90% retinal cells with retrograde labeling and non-specific staining (O, P) were in the negative region (third quadrant, both X and Y intensity <10^1^). Abbreviations: FL1-H: green fluorescence, 530/30 nm bandpass filter; FL2-H: orange fluorescence, 585/42 nm bandpass filter; FL3-H: red fluorescence, >650 nm bandpass filter.

**Figure 3:**
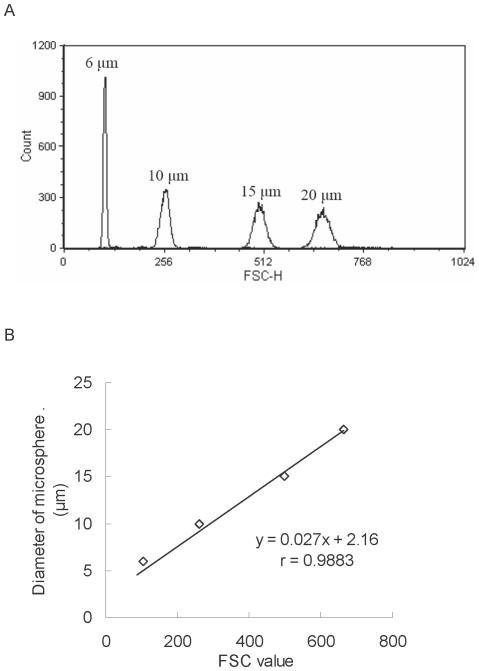
Flow cytometric size calibration. (A) Overlay of representative flow cytometric histograms of 6-, 10-, 15- and 20-µm-diameter microspheres. (B) Linear relationship between standard microsphere diameter and forward scatter (FSC) measurements. For each particle population, the mean FSC value was determined in triplicate.

To validate the distinguishability of three channels in one determination, three color beads (including FITC, PE, and PerCP-labeled beads) were used to adjust the setting condition of flow cytometer. Representative flow cytometric histograms of fluorescence-labeled beads are displayed in Fig. 2A–L. Among histograms of FITC-labeled bead, FL-1H histogram exhibited that peak of FITC-labeled bead (Peak_FITC_) was located on positive region (M2) (Fig. 2A), and FH-2H and FH-3H histograms exhibited that peak_FITC_ shifted to negative region (M1) (Fig. 2B, C). The same phenomena could be observed in histograms of the other two beads (Fig. 2D–F, G–I). When evaluating histograms of the mixture of three color beads, the following was noted. The peak_FITC_ heights of FL-1H, FL-2H and FL-3H histograms were in the ratio of 100∶35∶8 (Fig. 2A–C). The peak_PE_ heights of FL-1H, FL-2H and FL-3H histograms were in the ratio of 44∶100∶39 (Fig. 2D–F). The peak identification of three color beads-mixed histograms depended on position, peak height ratio and waveform. The FL-1H, FL-2H, and FL-3H histograms of the mixture of three color beads exhibited positive peaks of FITC, PE, and PerCP-labeled beads, respectively (Fig. 2J–L), suggesting the setting condition of flow cytometry could distinguish three fluorescence colors. To avoid background fluorescence and non-specific binding, two groups were used as negative controls [22], [23]: (I). retinal cells with retrograde labeling but without immunostaining (without primary and secondary antibodies) and (II). retinal cells with retrograde labeling and non-specific staining (fluorescence-conjugated secondary antibody alone). The results of flow cytometric dot plots showed that >99.98% group I cells (Fig. 2M–2N) and >99.90% group II cells (Fig. 2O, P) were in the negative region (third quadrant, both X and Y intensity <10^1^). These results suggested that background fluorescence and non-specific binding was only little effect on the measurement of positive cells. Commercial standard microspheres with various sizes underwent FSC measurements using the same setting condition in flow cytometry (Fig. 3A). The established flow cytometric method yielded acceptable precision for FSC determination with the coefficients of variation 0.20∼3.32% (data not shown). A linear equation was used to fit a calibration curve of microsphere diameter versus FSC value: *y = 0.027x+2.16* (eq. 2), where *x* = FSC value; *y* = microsphere diameter, with a unit of micrometer. The fitted equation had good linear relationship (r = 0.9883, Fig. 3B) to be applied for calculating the D_(v, Q)_ of RGCs.

### Correlation between the area diameter and apparent volume diameter of RGCs

**Figure 4:**
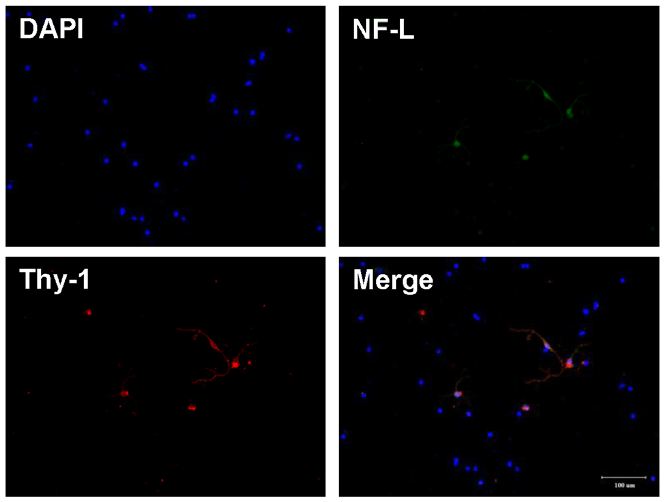
Fluorescence images of dissociated RGCs. Retinal cells were dissociated with papain, fixed with 4% paraformaldehyde in PBS, and stained with DAPI (blue). RGCs (arrowhead) were identified by double immunocytochemistry with anti-neurofilament (NF)-L antibody (green) and anti-thy-1 anitbody (red), bar = 100 µm.

**Figure 5:**
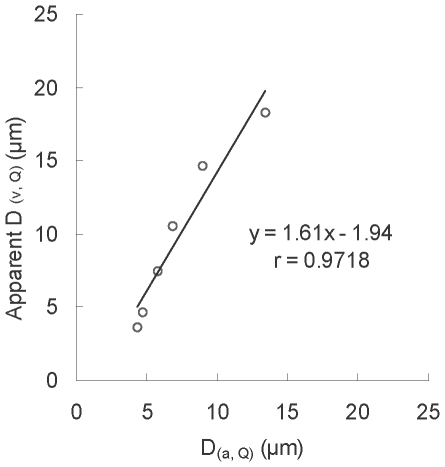
Linear relationship between area diameter (D_(a)_) and apparent volume diameter (D_(v)_) of normal RGCs. D_(a)_ was estimated by image-analysis software. Apparent (D_(v)_) was obtained by substituting its FSC value in the calibration curve of the standard microspheres (*y = 0.027x+2.16 x* = FSC value; *y* = diameter). (D_(a, 5)_, D_(v, 5)_), (D_(a, 20)_, D_(v, 20)_), (D_(a, 40)_, D_(v, 40)_), (D_(a, 60)_, D_(v, 60)_), (D_(a, 80)_, D_(v, 80)_), and (D_(a, 95)_, D_(v, 95)_) were plotted to obtain the equation: *y* = 1.61*x*−1.94. D_(v, Q)_ and D_(a, Q)_ represent the volume diameter and area diameter, respectively at the cumulative cell size percentile of Q in total RGCs.

After the treatment of RGC samples just prior to flow cytometric counting, these samples were also examined under a fluorescence microscope as the flatmount method (Fig. 4). The result of RGC size distribution was similar to the flatmount counting. It suggested that the RGC sample treatment before flow cytometric counting is not significantly affecting the size distribution of RGCs. The areas of RGCs (determined by image-analysis software) in the control group for Q at 5, 20, 40, 60, 80, and 95% were 15.14, 17.30, 25.95, 36.77, 62.72, and 140.57 µm^2^ corresponding to 4.39, 4.69, 5.75, 6.84, 8.94, and 13.38 µm (calculated by eq. 1) for the D_(a, Q)_ of RGCs, respectively. Flow cytometry revealed that the FSC values of RGCs in the control group for Q at 5, 20, 40, 60, 80, and 95% were 78, 104, 187, 290, 445, and 603 corresponding to 4.04, 4.86, 7.33, 10.28, 14.45, and 18.33 µm of apparent D_(v, Q)_ (calculated by eq. 2), respectively. There were −7.97% to 61.61% of differences between D_(a, Q)_ and apparent D_(v, Q)_ (Table 2). The relationship between D_(a, Q)_ and apparent D_(v, Q)_ is shown in Fig. 5. An linear equation (*y = 1.61 x−1.94*…eq. 3, r = 0.9718, *x*: D_(a, Q)_; *y*: apparent D_(v, Q)_) was fitted. The results indicated that RGC soma sizes determined from flatmount imaging and flow cytometry had good linear relationship. The sample preparing process of retinal cell suspension for flow cytometric determination did not obviously change RGC size distribution.

**Table 2:** Deviations of RGC size percentiles determined by flatmount and flow cytometry.

	Area (µm^2^)	Area diameter (D_(a)_, µm)	Volume diameter (D_(v)_, µm)	FSC value	Difference (%)
**Cumulative RGC percentage**					
Q5	15.14^a^	4.39^b^	4.04^c^	78^d^	−7.97
Q20	17.30	4.69	4.86	104	3.62
Q40	25.95	5.75	7.33	187	27.48
Q60	36.77	6.84	10.28	290	50.29
Q80	62.72	8.94	14.45	445	61.63
Q95	140.57	13.38	18.33	603	37.00
**RGC group**					
Small RGCs	15–70	4.37–9.44	5.09–13.26^e^	108–411^f^	-
Medium RGCs	70–125	9.44–12.62	13.26–18.38	411–601	-
Large RGCs	125-	12.62-	18.38-	601-	-

Difference (%) = (volume diameter−area diameter)/area diameter×100%.

a RGC area was obtained from the flatmount method by using an image-analysis software.

b D_(a, Q)_ was calculated from the circle area equation (eq. 1, 2× (area/3.1416)^1/2^).

c Apparent D_(v, Q)_: the RGC diameter was estimated by substituting its FSC value into the calibration curve of the standard microspheres (y = 0.027 x+2.16, eq. 2, *x* = FSC value; *y* = diameter).

d FSC value was obtained from the flow cytometric measurement of RGCs.

e D_(v, Q)_: the RGC diameter was estimated by substituting D_(a, Q)_ into the linear equation.

f Calculated FSC value: the FSC value was calculated from D_(v)_ (eq. 2).

### Effects of NMDA on survival rate and health of retinal cells

**Figure 6:**
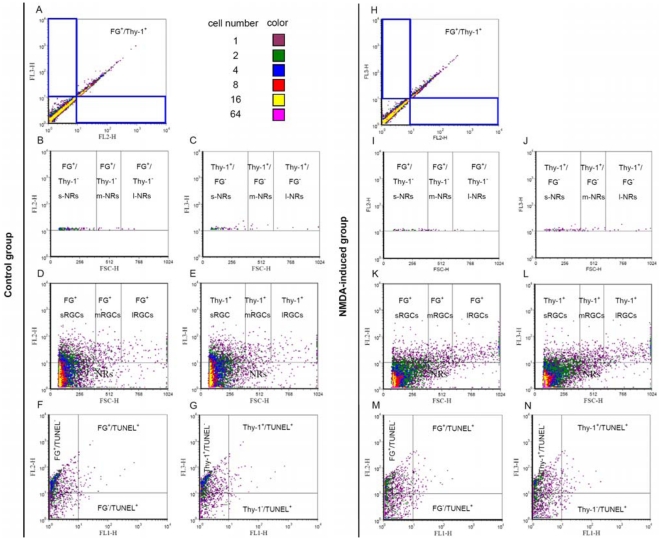
Flow cytometric analysis of triple-labeled retinal cells. Representative flow cytometric dot plots of triple-labeled (TUNEL, FG, and thy-1) cells of control group (A–G) and NMDA-induced group (H–N). RGCs and non-RGCs were evaluated by FG vs thy-1 labelings (A, H). Size of gated FG^+^/thy-1^−^ (gate 1) and FG^−^/thy-1^+^ (gate 2) cells were evaluated by FSC vs FG labeling (B, I) and FSC vs thy-1 labeling (C, J), respectively. Small, medium and large RGCs were evaluated by FSC vs FG labeling (D, K) and FSC vs thy-1 labeling (E, L). Apoptotic RGCs were evaluated by TUNEL vs FG labelings (F, M) and TUNEL vs thy-1 labelings (G, N). Abbreviations: FL1-H = TUNEL; FL2-H = Fluoro-Gold; FL3-H = Thy-1; FSC-H = Forward scatter; s-, m- and l-NRs = small, medium and large non-RGCs; s-, m- and l-RGCs = small, medium and large RGCs.

The effects of NMDA on retinal cells were further evaluated by flow cytometry. Representative flow cytometric dot plots of triple-labeled retinal cells are displayed in Fig. 6. In control group, FG^−^/thy-1^+^ cells (gate 1), which were considered to be non-RGC cells, comprised 5.31±2.66% of the total thy-1^+^ cells; FG^+^/thy-1^−^ cells (gate 2), which were also considered to be non-RGC cells, comprised 2.05±0.44% of the total FG^+^ cells (n = 6, Fig. 6A). In NMDA group, FG^−^/thy-1^+^ cells comprised 6.08±1.11% of the total thy-1^+^ cells; FG^+^/thy-1^−^ cells comprised 2.53±0.36% of the total FG^+^ cells (n = 6, Fig. 6H). FG/thy-1 double positive cells were considered to be RGCs [24]. Furthermore, RGCs were classified into three groups according to the projecting area of the RGC soma. The area was sequentially converted into area diameter (eq. 1), true volume diameter (eq. 3), and FSC values (eq. 2) as described in Table 2. The determined FSC values were then used to classify three-group RGCs and non-RGC in flow cytometry. The percentages of three-size FG^+^/thy-1^−^ cells among each size of FG^+^ cells were all <6.59% (n = 6, Fig. 6B, D) and three-size FG^−^/thy-1^+^ cells among each size of thy-1^+^ cells were all <5.34% (n = 6, Fig. 6C, E). After NMDA treatment, the number, FG intensity and thy-1 intensity of three-size non-RGC did not have any significant differences (n = 6, P>0.05, Fig. 6I, J) compared to control group. These results suggested that non-RGCs are only a minor effect on RGC quantification and damage assessment.

**Figure 7:**
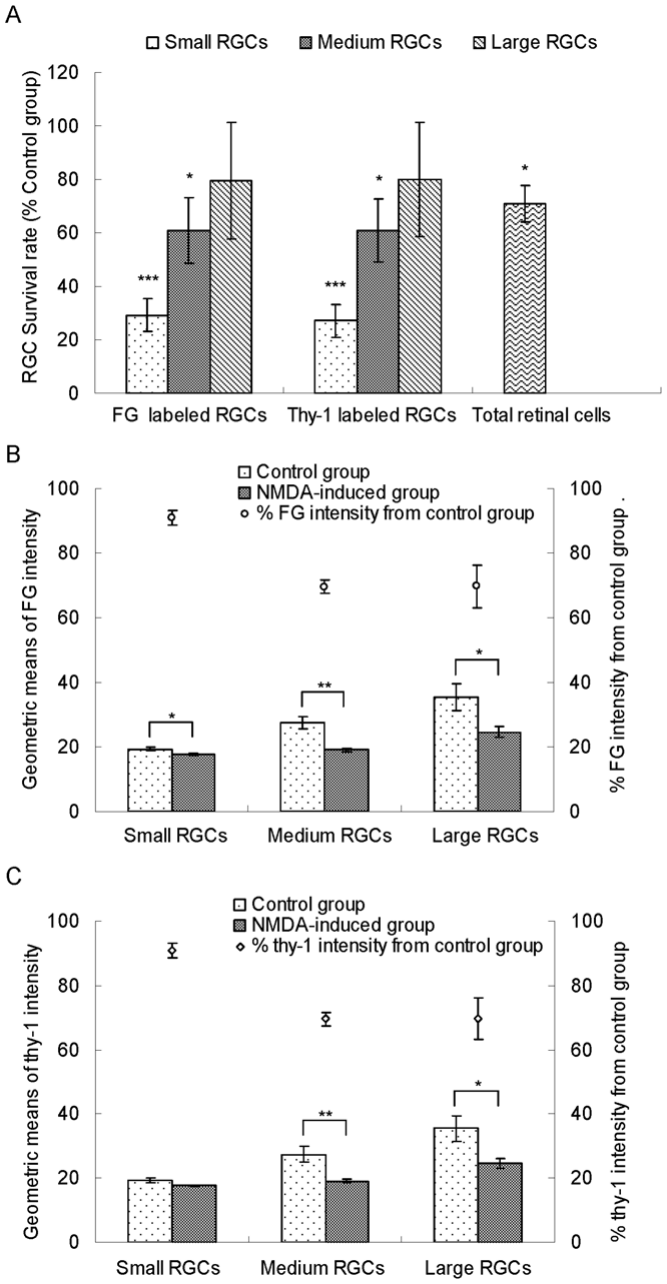
Quantitative flow cytometric analysis of FG and thy-1 labeled RGCs. (A) Effect of NMDA on survival rates of different size RGCs and total RGCs compared to the control group (n = 6). Total RGCs were counted using a hemacytometer and the percentages of different size RGCs in total retinal cells were determined by flow cytometry. The numbers of different size RGCs were calculated according to the above results. (B) Quantitative analysis of FG intensities of different size RGCs following intravitreal NMDA injection (n = 6). (C) Quantitative analysis of thy-1 intensities of different size RGCs following intravitreal NMDA injection (n = 6). FG and thy-1 intensities were expressed as geometric means calculated by CellQuest Pro software. Data were presented as mean ± standard error of the mean. Differences between the control and NMDA groups were analyzed using the Student's *t*-test. *P<0.05, **P<0.01 and ***P<0.001.

Flow cytometry confirmed that NMDA treatment resulted in a significant loss of small RGCs (58.74±4.35% in FG^+^ and 61.89±4.24% in thy-1^+^ RGCs, P<0.001) but not large RGCs (n = 6, Fig. 6K, L, Fig. 7A). Flow cytometry further showed that the geometric means of FG intensity of normal small, medium, and large RGCs were 19.39±0.57, 27.40±1.85 and 35.44±4.01, respectively. The coefficients of variation in FG intensity were good precision but slightly higher as increasing the size of RGCs. Following NMDA treatment, the percentages of the geometric means of FG intensities for the three groups of live RGCs were all significantly reduced (small: 90.96±2.24%, P<0.05; medium: 69.62±2.11%, P<0.01; and large 69.68±6.48%, P<0.05) as shown in Fig. 6K and Fig. 7B. After seven days of NMDA treatment, the protein levels of thy-1 in medium and large live RGCs were also significantly reduced. In comparison with the control group, medium and large live RGCs expressed 69.07±2.98% (P<0.05) and 69.91±6.23% (P<0.05, n = 6, Fig. 6L, Fig. 7C) thy-1 levels, respectively. Seven days after NMDA treatment, the number of TUNEL^+^ RGCs did not show any significant differences (Fig. 6F, G, M, N).

## Discussion

While flow cytometry is the most widely used method for determining cell number, cell size, and fluorescence intensity, the method has not yet been employed in the evaluation of the overall characterization of RGCs. The present study describes a novel flow cytometric method to simultaneously evaluate the viability and damage of large, medium, and small RGCs in a single determination.

Some theories suggest that RGC axon degeneration and somatic loss are sequential events. The pathological process might be initiated by axon damage which consequently impairs the retrograde transport pathway of neurotrophins leading to the death of RGCs [25], [26]. To identify RGC pathological conditions, various fluorescent tags have been used. In this study, retrograde transport of a fluorescent dye and an immunostaining technique that labeled RGCs and terminal uridine deoxynucleotidyl transferase dUTP nick end labeling (TUNEL) were used for detecting apoptotic cells. FG, a small-molecule fluorescent dye with a molecular weight of 472, remains stable for more than two weeks in stained neurons. In addition, FG has a commercially available antibody for enhancing the visualization of stained targets to avoid background interference in flow cytometric analysis [22]. Hence, FG was chosen as a tracer for retrograde labeling via the intact axons of RGCs. In turn, thy-1 is a specific marker for RGCs. The marker is used to quantify RGCs via immunohistochemistry and evaluate the health of RGCs under toxic damage by measuring thy-1 mRNA [12], [15], [27]. Thy-1-CFP (cyan fluorescent protein) transgenic mouse is an established animal model which has been studied for simplifying the quantification of RGCs [24], [28]. The available data suggest that thy-1 is a suitable surface marker for identifying RGCs and assessing RGC damage. In studying RGC toxic damage, some researchers use reverse transcriptase PCR or western blot to measure the level of total thy-1 mRNA or protein, respectively, was used as a serrogate marker for total RGC status [12], [15], [16]. Compared to previous studies, one advantage of the present study is that the investigated flow cytometry technique is capable of measuring thy-1 protein at the single-cell level. This advantage is attributable to the flow cytometric capability of allowing a single cell to be measured and determining fluorescent tag concentrations as low as 10–100 pM [10].

The flow cytometric FSC signal of cell had been reported to be proportional to cell volume. Apparent cell size can be obtained from the FSC signal-volume calibration curve of known size microspheres. Because the refractive index, surface features and shape of particles, are also important factors to contribute to the FSC signal, thus, the results of cell size determined from the flatmount and flow cytometric methods might not be equal for further explaining the deviation of measured cell size between two methods. During well-controlled experimental condition, the size distribution of rat RGCs was not changed in sample treatment process. Therefore, the size of FG-labeled RGCs was measured via microscopic imaging within a certain percentile which was also used to apply in flow cytometric RGC sizing for dividing RGCs into small, medium and large groups.

Rat RGCs were often classified by morphological analysis. However, published data about the soma size distribution are inconsistent [5], [8], [29]. The reasons for the inconsistency are unclear, but could be due to labeling method (specific or non-specific) and dye (well cellular uptake or not). The combination of retrograde labeling and thy-1 immunostaining could exclude non-specific cellular labeling. Thy-1^+^/FG^−^ and Thy-1^−^/FG^+^ cells, which were considered to be non-RGC cells, were endothelial cells and secondary FG-stained cells, respectively. Most Thy-1^+^/FG^+^ cells were RGCs, only few Thy-1^+^/FG^+^ cells were activated microglial cells. Two kinds of Thy-1^+^/FG^+^ cells could be further distinguished by morphology or cell markers (such as neurofliment-L). Earlier studies indicated that thy-1^+^/DiA^−^ cells are only 3% of total thy-1 positive cells in transgenic mice, and activated microglial cells are about 1.5% of total FG positive cells in rat. [24], [30]. Our results together with pervious studies, suggest that non-RGCs have only a minor effect on RGC quantification. It has been reported that acute RGC shrinkage only occurs during RGC apoptosis and apoptotic RGCs are rapidly cleaned up from the retina [31]. In addition, some rat studies show that the number of TUNEL-positive RGCs or apoptotic cells peaks at 24 hours and decreases to baseline by 72 hours after intravitreal NMDA injection [18], [19]. Our flow cytometric data TUNEL assay showed that there were no significant differences between the control and NMDA groups after seven days following intravitreal treatment. Taken together, these results indicated that apoptotic RGCs were rapidly cleaned up and RGC shrinkage was not a significant factor on three groups of RGC counts at 72 hours after NMDA administration. The inference awaits further investigation.

The hypothesis that there is a selective loss of RGC subtypes under glaucomatous stress is widely debated. For example, some studies report that small RGCs are lost after axotomy in adult rats [32]. Retinal flatmount and cross-sectional studies indicate that large RGCs are primarily lost in humans and monkeys with elevated intraocular pressure (IOP) [33], [34], [35]. In the present study, RGC quantitation by both flow cytometry and retinal flatmount showed the selective loss of small RGCs in the face of NMDA toxicity. These disparities could be attributed to different experimental conditions, including the sources of insults and animal species. Surprisingly, our flow cytometric data showed that the changes in thy-1 level had opposite results to RGC loss in NMDA toxicity. Previous studies involving psychophysical and electrophysiological examinations failed to support the hypothesis that there is selective dysfunction of large RGCs in patients with elevated IOP [36], [37]. As for the discrepancy between anatomical and functional data, studies addressing retinal structure-function relationships help rationalize the discrepancy as researchers have used two methods to analyze retinal structure and function in glaucoma. Analyses showed that the level of RGC dysfunction (or toxic damage) is more serious than structural damage, such as retinal shrinkage or RGC loss, suggesting that RGC dysfunction appears early. In other words, there is a lag time between RGC dysfunction and RGC loss [12], [13], [38], [39]. Previous studies indicated that elevated IOP caused both large (M cell) and small RGC (P cell) dysfunction [38], [39]. Thus, if the lag time of large RGCs is shorter than that of small RGCs, selective loss of large RGCs may be detected at the later stage. Our flow cytometric method showed that small RGCs were primarily dying during NMDA toxicity, but surviving small RGCs had less stress damage than the large RGCs. This may be because the lag time of large RGCs is longer than that of small RGCs in NMDA toxicity.

Another explanation involves the threshold in apoptosis for the three groups of RGCs. The thy-1 levels of normal RGCs were not lower than threshold and the damaged RGCs, below the threshold, had disappeared within initial 72 hours as their fluorescence could not be observed or determined in our study. In addition, previous studies have shown that the percentage change of thy-1 level or RGC function is larger than the fraction of RGC number loss in models of retinal damage [12], [15], [38], [39]. This information indicates that the majority of live RGCs were equal or higher than the threshold but that one portion of the live RGCs was dysfunctional. Thus, if most large RGCs are lost and most small RGCs are dysfunctional, a discrepancy between anatomical examination and functional examination in the elevated IOP model will exist. Our flow cytometric analysis, however, showed that large RGCs had greater mean fluorescence intensities of FG and thy-1 than small RGCs indicating that large RGCs appear more resistant to loss than small RGCs. This finding was consistent with the NMDA model but was different from the elevated IOP model. It is possible that large RGCs have a smaller surface-to-volume ratio causing a more significant loss when IOP is elevated [35]. Overall, the hypotheses suggest that the lag time or fluorescence intensity of markers and their level change depend on both the RGC population and the sources of insults. In addition, it is necessary to evaluate both RGC structure and function to obtain their relationships. The flow cytometric method described herein evaluates the viability and damage of large, medium, and small RGCs in a single determination.

The prevailing opinion regarding the primary site of damage in glaucoma is the RGC axons located at optic nerve head. The loss of the RGC soma occurs after axon damage in long-term duration [25], [26], [40], [41]. In the present study, FG-labeled RGC loss and the change of fluorescence intensity correlated closely with that of thy-1-labeled RGCs, suggesting that the latency period between axon damage and RGC soma loss is very short in NMDA toxicity.

Even though the established flow cytometric method provides a plethora of real-time data regarding RGCs, the method has some limitations. One is that flow cytometry is only equipped with three channels which limits 3 fluorescent tags to be evaluated simultaneously. Another challenge is that the adjustment of fluorescence compensation has become more difficult when multiple color markers are used to label cells. In addition to fluorescence parameters, FSC is another parameter of flow cytometry which can be used to evaluate RGC size in real-time. Perhaps further research could use biomarkers to confirm the morphologic classification of the RGCs.

In conclusion, the established flow cytometric method provides a high-content analysis for differentiating the loss and damage among three groups of RGCs. The downstream effectors of apoptotic damage and death pathways in each RGC group, however, are unclear. In addition, RGC death is a complex process involving more than one toxic insult. Further research is needed to examine the effects of other toxic insults on RGC groups by the established flow cytometric method and to explore possible downstream effectors of apoptotic damage and death pathways in each RGC group. This understanding may improve the treatment of pharmacological neuroprotection.
